# Exploring Biomarkers for Malaria: Advances in Early Detection and Asymptomatic Diagnosis

**DOI:** 10.3390/bios15020106

**Published:** 2025-02-12

**Authors:** Jacko Abiwaqash Harmonis, Sri Agung Fitri Kusuma, Yaya Rukayadi, Aliya Nur Hasanah

**Affiliations:** 1 Department of Pharmaceutical Analysis and Medicinal Chemistry, Faculty of Pharmacy, Universitas Padjadjaran, Jalan Raya Bandung-Sumedang KM 21 Jatinangor, Bandung 45363, Indonesia; jacko19001@mail.unpad.ac.id; 2 Department of Pharmaceutical Biology, Faculty of Pharmacy, Universitas Padjadjaran, Jalan Raya Bandung-Sumedang KM 21 Jatinangor, Bandung 45363, Indonesia; s.a.f.kusuma@unpad.ac.id; 3 Department of Food Science, Faculty of Food Science and Technology, Universiti Putra Malaysia, Serdang 43400, Selangor, Malaysia; yaya_rukayadi@upm.edu.my

**Keywords:** malaria, biomarker, *plasmodium*

## Abstract

Malaria is a tropical disease caused by the *Plasmodium* parasite, which was responsible for 249 million cases worldwide in 2022. Malaria is currently diagnosed using RDTs, PCR-based methods, or blood smear microscopy. Ideal biomarkers have been identified for malaria, with the potential for improving treatment, diagnosis, and overall clinical outcomes. This review discusses the types of existing biomarkers and the opportunities for new biomarkers to be used as diagnostic components in detecting *Plasmodium*, including in terms of sensitivity, detection limit, specificity, and the species of *Plasmodium* that can be detected. Following a comparison, five main ideal malaria biomarkers were identified, namely HRP2, pLDH, hemozoin, aldolase, and pGDH. These biomarkers distinguished themselves markedly from the others in terms of specificity in *Plasmodium* detection, sensitivity in analysis, and the use of non-invasive samples. Several other biomarkers, such as CRP, Ang-1, Ang-2, and PCT, show potential for malaria detection in terms of their ability to differentiate disease severity, and the levels of these biomarkers can be determined in the body for comparison with malaria parasitemia. Of the five ideal biomarkers, hemozoin and aldolase can still be developed regarding the types of samples used and their sensitivity to different *Plasmodium* species. Further research on the biomarkers CRP, Ang-1, Ang-2, and PCT is still needed to evaluate their potential.

## 1. Introduction

*Plasmodium* parasites are the causative agents of malaria, a tropical disease ranked among the most serious infectious diseases globally. Malaria cases worldwide increased by 5 million during 2021, reaching 249 million in 2022. It is estimated that malaria was responsible for 608,000 deaths in 2022 [1]. Malaria is a potentially fatal protozoan disease that arises from transmission of the *Plasmodium* parasite to humans through the bite of infected female Anopheles mosquitoes [2]. *Plasmodium malariae* (PM), *Plasmodium falciparum* (PF), *Plasmodium ovale* (PO), *Plasmodium vivax* (PV), and *Plasmodium knowlesi* (PK) are five species of the genus *Plasmodium* that are known to infect humans and cause malaria [3]. It is estimated that 99.7% of malaria cases are caused by the most prevalent and dangerous malaria parasite, *P. falciparum*, which is commonly associated with serious illness and death in the WHO African Region, particularly in Sudan [4]. The majority of deaths in the WHO Eastern Mediterranean Region are due to *P. falciparum*, which is responsible for around 90% of cases and has a higher case fatality rate than *P. vivax* [5].

The initial symptoms of malaria, which are similar to the flu and are present in all malaria species, are nonspecific [6]. Headaches, muscle aches, fatigue, malaise, nausea, fever, and vomiting are clinical signs of uncomplicated malaria. The symptoms of malaria, a feverish disease, typically appear 10 to 15 days following a mosquito bite [7,8]. Physicians who infrequently encounter malaria may mistakenly diagnose it as influenza, gastroenteritis, dengue, viral hepatitis, typhoid fever, or encephalitis due to the unpredictable and nonspecific clinical manifestations [6]. Specific diagnostic techniques are needed to help differentiate malaria from other feverish diseases. Early malaria detection can reduce disease severity and prevent progression [9,10]

Malaria is diagnosed using rapid diagnostic tests (RDTs) [11], polymerase chain reaction (PCR)-based methods [12], or blood smear microscopy [13]. In regions where malaria is prevalent, PCR-based techniques have not been widely adopted due to their complexity and expense [14]. Malaria RDTs are useful diagnostic instruments since cases may be diagnosed within 15 to 30 min, and there is no requirement for complex PCR equipment or trained experts [15]. Malaria parasites produce specific proteins called antigens in the blood of infected individuals, which are detected using malaria RDTs. Some RDTs detect only a single species (*P. falciparum* or *P. vivax*), whereas others are capable of detecting several species (*P. falciparum*, *P. vivax*, *P. malariae*, and *P. ovale*) [16]. However, the World Health Organization (WHO) and others claim that the currently available malaria RDTs have a number of drawbacks, including poor specificity, the inability to quantify parasite density, susceptibility to heat and moisture, and higher costs compared to microscopy-based techniques [15,17].

Biomarkers specific to malaria can be applied in malaria detection, for which many methods have been developed based on approaches involving a variety of biomarkers. Each biomarker generally varies in its sensitivity, specificity, and the types of samples that can be used to detect *Plasmodium*-causing malaria. To date, there are no reviews on the currently available malaria biomarkers used in the detection, including their sensitivity, detection limit, specificity, and the species of *Plasmodium* causing malaria that can be detected. Therefore, this review summarizes all potential biomarkers for malaria detection, starting from the life cycle of the malaria parasite, its virulence factors, and biomarkers that have already been investigated. Hopefully, this review can serve as a reference for researchers carrying out malaria detection, facilitating the selection of detection methods for analyzing certain specific malaria biomarkers.

## 2. The Life Cycle of Malaria Parasite

The malaria parasite has a complex life cycle involving a vertebrate host and the Anopheles mosquito. In the initial phase of infection, sporozoites in mosquito saliva enter the human host’s bloodstream after epidermal penetration before migrating to hepatocytes to initiate an asexual replication process [18]. Thousands of merozoites are released during this phase (also known as the hepatic or pre-erythrocytic phase) when infected hepatocytes rupture [19].

**Figure 1:**
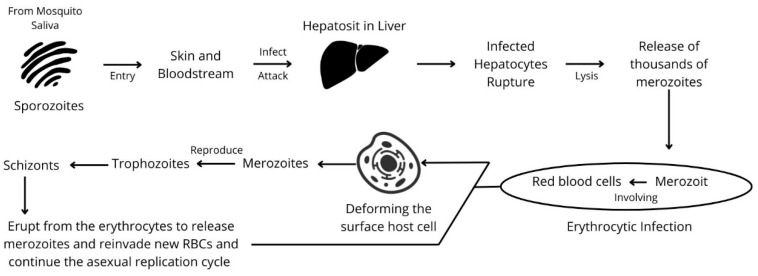
The life cycle of malaria.

During erythrocytic infection, merozoites and red blood cells (RBCs) interact. *Plasmodium* parasites cause erythrocytic infections by producing proteins that bind to red blood cell receptors. *Plasmodium* parasites can undergo antigenic variation and adhere to the endothelial cells lining blood vessels (sequestration). The merozoite head aligns itself with the erythrocyte membrane by bending the surface of the host cell. To undergo a second round of asexual reproduction, the parasite then enters the erythrocyte and triggers cytoskeletal rearrangement in response to the infection. Following their invasion of RBCs and to continue the asexual replication cycle, merozoites divide into trophozoites and schizonts, which leave the erythrocytes to release additional merozoites and infiltrate new RBCs [19,20]. A summary of the malaria life cycle can be seen in Figure 1.

## 3. Pathogenesis of Malaria

In *P. falciparum* malaria, knobs or protuberances appear on the surface of infected erythrocytes 12 to 15 h after invasion. Mature parasite-containing red blood cells are sequestered by adherents into critical organs, including the brain, and obstruct metabolism, microcirculatory flow, and vascular endothelium function [21].

Red blood cells are specifically infected by *P. vivax*, *P. ovale*, and *P. malariae* (for example, *P. vivax* only infects young red blood cells), and parasitemia levels are often less than 1%. *P. falciparum* and *P. knowlesi* can develop extremely high parasite numbers and are less selective. In contrast, *P. vivax* causes infected red blood cells to become enlarged and deformed. As the *P. falciparum* parasite matures, infected red blood cells become more round and rigid [22,23].

In response to malaria, the host increases splenic immune activity and clearance via filtration, hastening the elimination of infected and parasitized erythrocytes [24]. Fever and other pathological effects are caused by the schizont ruptures, with parasite and host cells released into the bloodstream. Pro-inflammatory cytokines are released as a result of this stimulation of monocytes and macrophages [21,25].

## 4. Virulence Factors

**Figure 2:**
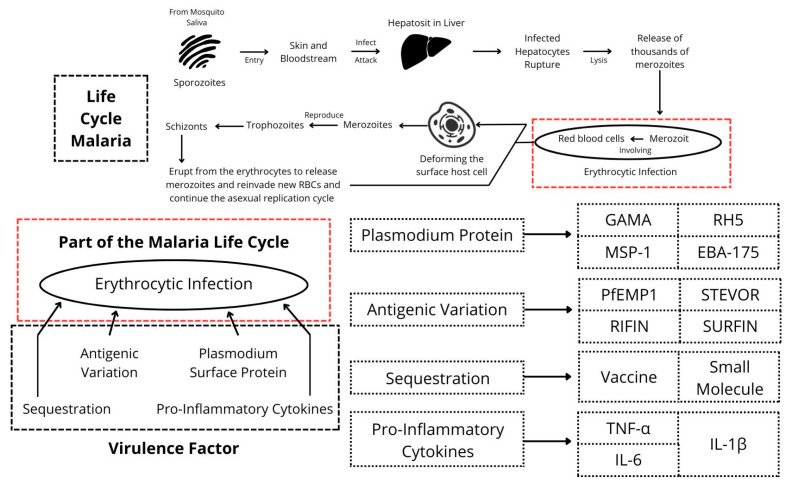
Virulence factors of malaria.

Virulence factors are characteristics or properties of a pathogen that enhance its capacity to spread illness. A number of virulence factors, including sequestration, pro-inflammatory cytokines, antigenic variation, and *Plasmodium* surface protein, influence the severity and progression of malaria [19,20,21,25]. Details of the virulence factors of malaria can be seen in Figure 2.

### 4.1. Plasmodium Surface Protein

*Plasmodium* surface proteins are crucial for the malaria parasite’s life cycle and its interaction with the host [26]. *Plasmodium* has various surface proteins that play essential roles in host cell invasion, such as glycosylphosphatidylinositol-anchored micronemal antigen (GAMA), reticulocyte-binding protein homolog 5 from *P. falciparum* (PfRH5), merozoite surface protein 1 (MSP-1), and erythrocyte-binding antigen 175 (EBA-175). GAMA facilitates the invasion of red blood cells by interacting with receptors on their surface [27]. In every strain that has been investigated, PfRH5 was shown to be essential for erythrocyte invasion [28]. MSP-1 is the most common protein on the surface of the *Plasmodium* merozoite that causes malaria and infiltrates erythrocytes [29]. During the invasion, the *P. falciparum* protein EBA-175 binds glycophorin A, the main glycoprotein present in human erythrocytes [30].

### 4.2. Antigenic Variation

*Plasmodium* parasites can undergo antigenic variation, changing the surface antigens on infected red blood cells. This helps the parasite evade the host immune system, making it challenging for the immune system to mount an effective response [31]. For instance, the multigene families encode highly polymorphic antigens PfEMP1, RIFIN, STEVOR, and SURFIN in *P. falciparum* that exhibit a significant amount of antigenic variety to facilitate immune evasion [32]. PfEMP1 is a highly variable protein that is the primary antigen produced on the surface of infected red blood cells. It is also the primary virulence factor and a key target of naturally acquired immunity [33]. Antigenic variation is connected with immune system evasion. Thus, antibodies are not produced against *Plasmodium* surface protein, preventing this virulence factor of malaria from potentially being utilized as an effective biomarker for malaria detection. Further study is still needed to support this statement, as no research has yet been conducted regarding the antigenic variation in biomarkers.

### 4.3. Pro-Inflammatory Cytokines

Cytokine dysregulation is said to be a significant molecular indicator of the cell-mediated immune response during malaria infection. This dysregulation can lead to an excessive inflammatory response, contributing to disease severity [34]. Pro-inflammatory cytokines such as tumor necrosis factor-alpha (TNF-α), interleukin-1β (IL-1β), and interleukin-6 (IL-6) are produced by immune cells when *Plasmodium* parasites infect and multiply inside red blood cells. In order to control the infection, these cytokines and other mediators elicit an inflammatory response [35]. To date, three studies have examined whether these pro-inflammatory cytokines can be used as biomarkers [36,37,38]. However, none of these studies confirmed their potential as biomarkers, although further studies are still needed for a decisive conclusion.

### 4.4. Sequestration

Sequestration refers to the ability of infected red blood cells to adhere to the endothelial cells that line blood vessels. This aids in the pathophysiology of severe malaria by preventing the parasite from being cleared by the spleen [39]. Sequestration is directly impacted by host endothelium proteins (receptors and adhesins) and parasite proteins (ligands). It is anticipated that vaccines or small-molecule inhibitors that prevent sequestration will aid in the development of novel approaches that either prevent or alleviate sickness [39,40,41].

## 5. Biomarkers

The molecules that may be subjectively quantified and evaluated as a sign of pathogenic stress, biological processes, or a treatment response are known as biomarkers [42]. Biomarkers are used to track disease development. Prior to diagnosis, this involves risk assessment and screening. During diagnosis, they help determine the stage, classification, and selection of first-line treatment. Once diagnosis is complete, biomarkers are used to monitor treatment, select additional treatments, and monitor for recurrent disease [43,44]. Targeting biomarkers can enhance treatment, prognosis, and diagnosis. Finding the right biomarkers is essential for customized medicine and improved overall clinical outcomes [44].

Molecular, cellular, and imaging biomarkers are the three categories of biomarkers. Molecular biomarkers are measurable markers derived from proteomic and genomic methodologies. Biological samples such as blood, plasma, bronchoalveolar lavage fluid, cerebrospinal fluid, and biopsies can all be used to detect this type of biomarker [45,46]. Molecular biomarkers comprise a wide range of molecules, including lipid metabolites, proteins, peptides, nucleic acids (DNA and RNA), and others, both big and small [44].

Clinical and laboratory examinations can use cellular biomarkers, which are biological and quantifiable signs. In order to determine a patient’s prognosis or likelihood of responding to a certain treatment, cellular biomarkers are frequently examined and assessed in soft tissue, bodily fluids, or blood [47,48].

Imaging biomarkers are traits that can be measured and evaluated objectively and are used as markers of biological processes, normal pathological processes, or pharmacological reactions to therapeutic interventions. Magnetic resonance imaging (MRI), computed tomography (CT), and positron emission tomography (PET) are the three categories of imaging biomarkers [49].

The characteristics of an ideal biomarker for diagnosis include the following:

### 5.1. Specificity and Sensitivity

A good biomarker should be specific to the particular condition or disease it is intended to identify. Thus, it should not be influenced by other factors or conditions [50]. Sensitivity refers to the ability of a biomarker to detect the presence of a disease when it is truly present. A highly sensitive biomarker can accurately identify individuals with the disease [51].

### 5.2. Reliability

Biomarkers should provide accurate, precise, reliable, and reproducible results. This ensures that the information obtained from the biomarker is trustworthy, facilitating correct diagnosis and appropriate medical decisions [44,52].

### 5.3. Cost-Effectiveness

Biomarkers should be economically feasible for routine use in clinical practice. Widespread adoption can be limited by the high costs associated with testing and analysis [53].

### 5.4. Easily Measured

A biomarker should be quantifiable using standardized and reproducible measurement techniques. This allows for precise monitoring of changes in biomarker levels over time and facilitates comparison across different studies and laboratories [54].

### 5.5. Non-Invasiveness

Whenever possible, non-invasive methods of sample collection (e.g., blood, urine, and saliva) are preferred over invasive procedures. Non-invasive biomarkers are more likely to be accepted by patients and healthcare providers [55].

## 6. Biomarkers for Malaria

Early detection of specific *Plasmodium*-mediated infection biomarkers can be essential for developing illness treatment plans and selecting the optimal prophylactic measures [56]. Biomarkers used in malaria detection generally include antigens, enzymes, and proteins secreted or produced by various *Plasmodium* species. The type of malaria biomarker determines the information obtained in the diagnostic process. Molecular biomarkers primarily include genetic and protein markers that indicate the presence of the malaria parasite. For instance, *Plasmodium* DNA or RNA can be detected using polymerase chain reaction (PCR), providing highly sensitive and specific identification of the parasite [57]. Beyond molecular markers, cellular biomarkers play a crucial role in malaria diagnosis, reflecting the host immune response and cellular changes caused by infection. The presence of parasitized red blood cells (pRBCs) in a blood smear is a fundamental diagnostic feature, typically identified through microscopy [58]. Imaging biomarkers, though less commonly used in routine diagnosis, provide valuable insights into malaria pathophysiology [59].

The malaria-associated biomarkers currently used to identify disease are briefly explained below.

### 6.1. Histidine-Rich Protein 2 (HRP2)

The biomarker histidine-rich protein 2 (HRP2) is widely employed in the development of diagnostic methods for malaria. *Plasmodium falciparum* produces the protein HRP2, which is distinct to this species [58]. A mosquito bite causes the water-soluble protein HRP2 to be released from infected erythrocytes and enter the cytoplasm of red blood cells. The digesting vacuole, food vacuole, and membrane surface of infected red blood cells all contain HRP2 [58]. HRP2 is the foundation of many modern RDT assays and has been utilized as a biomarker for *Plasmodium falciparum* infection [60]. RBC cytosolic components, such as HRP2, are released into the bloodstream when parasites break out from the host cell. HRP2 in plasma can reach 100 μg/mL [58]. One major challenge in the application of HRP2 in the detection process results from genetic deletion of the pfHRP2 and pfHRP3 genes in some strains of *P. falciparum* [61]. In such cases, HRP2, which is essential for detection in standard RDTs, is absent. This means that the RDT may fail to detect HRP2 even if a person is infected with *P. falciparum*, leading to false negative results. This genetic variation, although not universal, is becoming more common in certain regions and thus a growing concern for malaria diagnosis [61,62].

If detection is desired, HRP2 can be found in the serum, plasma, cerebrospinal fluid, and urine of infected patients [63]. It can be concluded that HRP2 is a specific biomarker for the detection of *Plasmodium falciparum*. HRP2 detection based on rapid diagnostic tests (RDTs) has been used in malaria diagnostics [64]. HRP2 can thus be classified as an ideal biomarker for use in detecting malaria, based on the use of non-invasive samples to give specific and sensitive results for *P. falciparum*.

### 6.2. Plasmodium Lactate Dehydrogenase (PLDH)

*Plasmodium* lactate dehydrogenase (pLDH) is an enzyme found in various species of the *Plasmodium* genus that are responsible for causing malaria in humans [65]. pLDH is primarily found within RBCs infected by *Plasmodium*. This enzyme has a crucial role in the parasite’s energy metabolism by catalyzing the conversion of pyruvate to lactate [66]. In addition to being present within infected red blood cells, pLDH may also be released into the bloodstream upon rupture of infected red blood cells during the parasite’s replication cycle. This release of pLDH can contribute to its detection in various diagnostic tests for malaria [65]. Furthermore, there is a clear correlation between the level of parasitemia and the blood levels of pLDH. Because of these benefits, pLDH has been extensively studied as a potentially useful diagnostic antigen and incorporated into numerous RDTs for malaria [64,67]. pLDH is a biomarker that can be used to detect various *Plasmodium* species, which demonstrates that it is not specific to only a single species [64]. Blood is also a suitable sample for detection because pLDH can be released into the bloodstream when infected red blood cells rupture.

Thus, pLDH can be classified as an ideal biomarker for use in detecting malaria based on its advantages, where the pLDH levels in the blood are directly correlated with the level of parasitemia. In addition, non-invasive samples can be used to provide specific results for various *Plasmodium* species.

### 6.3. Hemozoin

When blood is digested, blood-feeding parasite species like *Plasmodium* spp. create hemozoin, an insoluble brown microcrystalline substance [58,68]. Hemozoin is a byproduct of the malaria parasite’s digestion of hemoglobin in RBCs and is typically found within the digestive vacuoles of the parasite [69]. Hemozoin is utilized as a malaria pigment in the development of antimalarial medications and diagnostic instruments [70,71]. As a biomarker, it can be used to detect various *Plasmodium* species [72]. Hemozoin is produced when parasites infiltrate the host red blood cells; its levels are correlated with the disease course, and it is found in gametocytes that transmit malaria [69].

Hemozoin can be used to detect malaria, with blood being a suitable sample for detecting the formation of hemozoin, but it is not specific for certain species of *Plasmodium*. Currently, RDTs have been developed based on hemozoin detection. Thus, hemozoin can be classified as an ideal biomarker for detecting malaria using non-invasive samples to provide specific results for *Plasmodium*.

### 6.4. Aldolase

In the life cycle of the malaria parasite, aldolase plays a crucial role in glycolysis, the process by which the parasite generates energy. In the glycolytic process, aldolase catalyzes the cleavage of fructose-1,6-bisphosphate to produce dihydroxyacetone phosphate and glyceraldehyde-3-phosphate [73,74]. Because aldolase demonstrates low sensitivity in the currently available quick diagnostic instruments, it is less frequently utilized [75]. The low antigen concentrations in infected erythrocytes are primarily responsible for this poor sensitivity [56].

RDTs based on the aldolase biomarker were developed in 2022 by Fitri et al. [16]. Aldolase is a biomarker that can be used to detect more than one *Plasmodium* species, with lower sensitivity but higher specificity in detecting *Plasmodium vivax* compared to other biomarkers [75,76]. Thus, aldolase can be classified as an ideal biomarker for use in detecting malaria based on its use of non-invasive samples to provide specific results for *P. vivax*. However, the disadvantage is the limited sensitivity for other *Plasmodium* species.

### 6.5. Plasmodium Glutamate Dehydrogenase (PGDH)

*Plasmodium* glutamate dehydrogenase (GDH) is an enzyme found in various species of the *Plasmodium* genus, which are the parasites responsible for causing malaria in humans [58]. The ubiquitous enzyme glutamate dehydrogenase is essential for both ammonium absorption and glutamate catabolism. The primary role of glutamate dehydrogenase is to use NADP(H) or NAD(H) as cofactors in converting L-glutamate into α-ketoglutarate and ammonia [77]. One prevalent malaria biomarker that is present during the parasite’s intra-erythrocytic cycle is *Plasmodium* glutamate dehydrogenase. Because the host red blood cells lack this enzyme, it is a potent biomarker [58,78]. PGDH can be classified as an ideal biomarker for use in detecting malaria based on the use of non-invasive samples, and it can be found in various *Plasmodium* species that cause malaria in humans.

### 6.6. C-Reactive Protein (CRP)

As a nonspecific biomarker of inflammation, C-reactive protein (CRP) indicates probable bacterial infection [79]. Several studies have found that CRP is a viable marker for evaluating the severity of malaria infections despite the fact that it is not a specific biomarker for diagnosing malaria infection [80,81,82]. Dengue and malaria infections exhibit comparable symptoms and cause comparable CBC parameter abnormalities. It has been noted that CRP is a helpful biomarker for differentiating between dengue and malaria [83]. It has been demonstrated that CRP levels and blood parasite density are significantly correlated in individuals regardless of whether they have clinical symptoms. In addition to attaching to contaminated erythrocytes and aiding in their removal, CRP is essential for the activation of platelets and the complement system [84].

CRP is not a specific biomarker for malaria detection but can be used to determine whether or not CRP levels are normal in patients. It is likely that patients with normal or reduced CRP levels do not suffer from malaria parasitemia. Therefore, normal CRP levels can be used to exclude malaria if microscopy and RDTs are not available [84]. Thus, CRP cannot be classified as an ideal biomarker for malaria detection due to its lack of specificity. However, determining whether its levels are normal can be useful and potentially connected to the level of malaria parasitemia.

### 6.7. Angiopoietin-1 (Ang-1) and Angiopoietin-2 (Ang-2)

The severity of malaria depends on the host–parasite relationship, and the parasite’s capacity to cytoadhere to red blood cells and the endothelium is a crucial component [85]. Endothelial cells express angiopoietins, which are ligands of the Tie-2 receptor that control endothelial quiescence during regular physiological processes [86]. Higher levels of Ang-1, which are stored in platelets, encourage quiescence in the vascular endothelium under normal physiological circumstances. An increase in the cytoadherence of infected erythrocytes to the vascular endothelium is primarily linked to the generation of angiogenic factors in *Plasmodium falciparum* malaria [87]. A secreted glycoprotein called Ang-2 promotes endothelial cell migration, survival, and proliferation while also disrupting the connection between endothelial cells and perivascular cells, thereby controlling vascular remodeling [87]. Ang-2 functions as a functional antagonist when Ang-1 is present, and the interaction between Ang-2 and Tie-2 blocks the anti-inflammatory, antiapoptotic, and protective effects of Ang-1 [87,88].

Ang-1 and Ang-2 cannot be classified as ideal biomarkers for use in detecting malaria because they lack specificity. However, there is evidence in the literature demonstrating that Ang-1 and Ang-2 have potential in malaria detection based on their association with increasing the cytoadherence of infected erythrocytes to the vascular endothelium.

### 6.8. Procalcitonin

The thyroid and adipose tissue are the primary producers of procalcitonin (PCT), the precursor of calcitonin hormone, which contains 116 amino acids [89]. The importance of PCT for *Plasmodium falciparum* malaria is well established. The majority of reports emphasize that severe malaria is associated with higher PCT levels than uncomplicated malaria [90,91]. Additionally, there is a strong link between parasitemia and PCT [92], and malarial mortality has been observed in several populations [91]. However, findings from earlier research on the link between severe malaria and PCT are inconsistent. This discrepancy regarding PCT’s function in severe malaria has been ascribed to a number of factors in the studies, including sample size, patient age, ethnicity, and inaccurate clinical classification of patients.

PCT cannot be classified as an ideal biomarker for malaria detection because it is not specific, considering the previous studies show that there are still inconsistencies regarding the use of PCT in detecting severe malaria. Thus, at present, PCT can only be used to compare categories of malaria, namely to differentiate severe from uncomplicated infections.

Table 1 is a summary of the biomarkers that can be currently used to detect malaria and the *Plasmodium* species that can be detected.

## 7. Current Biomarkers and Malaria Detection Method

### 7.1. HRP2

In the study by Akotet et al., 2013, malaria detection was carried out using the PfHRP2 rapid diagnostic test method. Using microscopy as the gold standard, 592 feverish individuals in Gabon, Central Africa, were diagnosed according to the malaria species. Sensitivities were 100% in younger children, 96.0% or higher for *Plasmodium falciparum*, and 62.5% for non-*P. falciparum* malaria species. The detection method used had a specificity of 96.6% and a false positive rate of 9.3%. Thus, the HRP2 rapid diagnostic test method accurately diagnoses *P. falciparum* infection [94].

In the study by Li et al., 2024, a microfluidic point-of-care (mPOC) immunoassay for the quick quantification of PfHRP2 was developed to meet the demand for straightforward, precise, and field-deployable malaria diagnostic assays. With a linear response ranging from 0 to 40 ng/mL, an R2 correlation coefficient of 0.995, and a lower limit of detection of 0.18 ng/mL, the ultrasensitive mPOC immunoassay is at least 20 times more sensitive than mRDTs. The mPOC immunoassay method is accurate in diagnosing *Plasmodium falciparum* infection. Until now, related research has not been carried out on other *Plasmodium* species. This method has the advantage of being much easier to perform, allowing its use by providers in primary healthcare centers with minimal training. However, it is limited to use with purified serum samples and requires long incubation times (≥1 h) [95].

### 7.2. PLDH

In the study of Pereira et al., 2015, a micellar aqueous two-phase system (ATPS) was used for increased sensitivity of LFA detection. To focus on and detect pLDH at the same time, LFA was incorporated into the 3-D design. The method uses immobilized goat anti-mouse IgG secondary antibodies specific to the primary anti-PLDH antibody. In contrast to a standard LFA configuration with a 10 ng/mL pLDH detection limit, the LFA with paper-based micellar ATPS successfully detected pLDH at 1.0 ng/mL, which corresponds to a 10-fold increase regarding the detection limit and resulted in a sensitive assay. The advantages of this method are that it is a cheap and rapid paper-based test that has the potential to detect infectious disease biomarkers in resource-limited settings. However, it is limited by its lower sensitivity compared to sophisticated laboratory-based tests [96]. However, the level (percentage) of sensitivity obtained through this detection method was not characterized.

In the study of Lee et al., 2012, a sensitive aptasensor for *Plasmodium* lactate dehydrogenase was developed that can be used to diagnose malaria. Tests were carried out on *P. falciparum* and *P. vivax*, with the LDH proteins of both selectively detected with a detection limit of 1 pM. Blood proteins for infection with *P. vivax* and *P. falciparum*, the two primary malaria species, were detected at low detection limits (1 parasite/μL). Related research has not been carried out on other *Plasmodium* species. The advantages of this method are intrinsic and include strong target affinity and selectivity, thermal stability, easy chemical synthesis and modification, and ease of discovery and analysis compared to antibodies [97].

### 7.3. Combination HRP2-PLDH

Kantor et al., 2021, developed the Antibody-free Dual-biomarker Rapid Enrichment Workflow (AnDREW) to purify PLDH and HRP2 from large blood samples (150 μL). The analytical sensitivity for HRP2 and PLDH detection was improved by 11 and 9 times, respectively, using the AnDREW-enhanced RDTs in comparison to the unenhanced RDTs. Additionally, the limit of detection for PLDH was improved by 11 times (3.80 parasites/μL) using the AnDREW-enhanced RDTs in comparison to the unenhanced RDTs (42.31 parasites/μL). The sensitivity obtained by PLDH and HRP2 increased before and after the unenhanced method, where the PLDH sensitivity ranged from 0.7456 to 6.605 based on the slope of the best-fit line, corresponding to a 9-fold increase. The sensitivity of HRP2 ranged from 1.162 to 13.25 based on the slope of the best-fit line, corresponding to an 11-fold increase. The advantages of this method are the use of cheaper eluents and that the eluted PLDH is not complexed with molecular recognition elements [98].

### 7.4. Combination HRP2-Aldolase

In the study by Akotet et al., 2013, malaria detection was carried out using the PfHRP2/pan aldolase combination rapid diagnostic test method. Using microscopy as the gold standard, 592 feverish individuals in Gabon, Central Africa, had malaria species diagnosed. The detection sensitivity was at or above 96.0% for *Plasmodium falciparum* and 62.5% for non-*P. falciparum* malaria species, with higher sensitivity in younger children (100%). The detection method used had a specificity of 87.3% and a false positive rate of 27.1%. Overall, 32.5% of all tests produced “faint bands”, and only two resulted from samples with parasitemia below 100 p/μL. The advantage of this method is it only detects live parasites. Furthermore, the aldolase gene is highly conserved. However, it is limited by the low prevalence of non-*P. falciparum* malaria infections [94].

### 7.5. Hemozoin

Yadav et al., 2022, describe the use of magnetic surface-enhanced Raman spectroscopy (M-SERS) to detect hemozoin, a metabolic byproduct of the malaria parasite that exhibits paramagnetic characteristics. Its advantage compared to measurements conducted on the traditional SERS substrates without any magnetic field is that the SERS signal intensity was increased tenfold. For M-SERS, the hemozoin detection limit was as low as 10−11 M (<10 parasites/µL), which allows its use in detecting malaria in its early stages. The sensitivity is higher than that of other traditional detection methods like laser desorption mass spectrometry and blood cell counting [99].

### 7.6. Aldolase

In the study by Wang et al. 2014, the monoclonal antibody (mAb) 1C3-12 F10 pair was chosen from a number of clones that secrete anti-aldolase mAb and produced exclusively for detecting the *P. vivax* aldolase antigen. The mAb 1C3-12 F10 combination, which targets aldolase unique to *P. vivax*, demonstrated exceptional sensitivity and specificity of 97.4% and 100%, respectively. Additionally, 100% and 99.5% positive predictive values (PPV) and negative predictive values (NPV) were noted. The advantage of this method is that it was developed to specifically detect only the *P. vivax* aldolase antigen [76].

### 7.7. PGDH

Singh et al., 2018, report on how to use a carbon dot coupled to a particular aptamer to detect *P. falciparum* glutamate dehydrogenase based on protein-induced fluorescence amplification. A linear correlation was found between the fluorescence intensity of the Cdots–aptamer conjugate and the concentration of PfGDH, with a limit of detection (LOD) of 0.48 nM and a dynamic range of 0.5 nM to 25 nM (R^2^ = 0.98). Using the technique on diluted serum samples, it was possible to identify PfGDH with a limit of detection of 2.85 nM [100].

### 7.8. CRP

Addai-Mensah et al., 2019, assessed how well high-sensitivity CRP (hs-CRP) could be used to diagnose and assess malaria morbidity in a pediatric population in Ghana. The study examined 167 cases of *Plasmodium* parasitemic patients and 100 microscopically confirmed nonmalarial parasitemic subjects as controls, corresponding to a total of 267 subjects. The median hs-CRP level was considerably higher for high malaria parasitemia than for both moderate and low malaria parasitemia. The sensitivity (96.4–97.4%) and specificity (79.3–93.1%) increased with rising hs-CRP cutoff (3.12–4.64 mg/L), with the exception of significant parasitemia, when a decrease in sensitivity (80.9%) was noted. The drawback of this study is that only microscopy was used for malaria diagnosis and not tests such as RDT or more sensitive diagnostic tools such as PCR [84].

The abovementioned malaria detection methods using various biomarkers are summarized in Table 2.

## 8. Conclusions and Future Perspectives

Based on our discussion, we found that there are five biomarkers currently in use that are ideal for application in malaria detection methods, namely HRP2, pLDH, hemozoin, aldolase, and pGDH. Other biomarkers, such as CRP, Ang-1, Ang-2, and PCT, have the potential for malaria detection based on the possibility of differentiating malaria severity and determining the levels of these biomarkers in the body and comparing them with malaria parasitemia. However, several shortcomings still exist, such as a lack of sensitivity and specificity for *Plasmodium*. Biomarkers are still being developed that may possibly become ideal and specific biomarkers for malaria.

Furthermore, RDTs have been developed for the five ideal malaria biomarkers. The advantages of RDT examination as a tool for rapid malaria diagnosis are that pathogen infection can be rapidly confirmed, there are no requirements for specialized knowledge and expensive equipment and the simplicity of the procedure.

Regarding the type of samples used for malaria biomarker detection, blood samples are generally the most abundant and suitable. This is related to the erythrocytic infection stage of the malaria parasite’s life cycle, during which virulence factors are produced. Malaria biomarkers are also produced in infected red blood cells during this stage.

Regarding currently applied methods for malaria detection, several have been developed from known biomarkers, where each biomarker gives different results in detection. This can be seen from the sensitivity and specificity resulting from the detection test. It is known that HRP2 when used as a biomarker, produces sensitive and specific results for *P. falciparum*. Such high sensitivity and specificity are the results of the HRP2 protein being unique to *P. falciparum* and therefore not found in other *Plasmodium* species. Regarding the pLDH biomarker, it is known that pLDH provides specific test results for various species of *Plasmodium*. The level of pLDH in blood is also correlated with the level of parasitemia. Hemozoin is produced in all parasite species, and hemozoin crystals are produced when parasites attack red blood cells whose contents are related to disease development. Therefore, hemozoin can be used as a biomarker for detecting various species of *Plasmodium*. However, there are currently limitations for determining which species of *Plasmodium* are detected from the test. There is still potential for further development and investigations regarding the *Plasmodium* species that can be detected based on hemozoin. Regarding its use as a biomarker, aldolase is known to allow sensitive and specific results for *P. vivax* detection. However, it has limited sensitivity if used for other *Plasmodium* species. Thus, it remains a challenge to obtain a specific and sensitive aldolase-based method for the detection of other *Plasmodium* species. Use of pGDH as a biomarker is based on this enzyme being found in all *Plasmodium* species. pGDH can provide specific test results for *P. falciparum*. CRP is a nonspecific biomarker and cannot be used to determine the species of *Plasmodium*. However, CRP can be used to assess whether levels are normal and compared with malaria parasitemia levels. Ang-1 and Ang-2 are known to have the potential for malaria detection based on increased cytoadherence of infected erythrocytes to the vascular endothelium. However, there are no studies demonstrating the detection of Ang-1 and Ang-2 in malaria. Thus, further research is needed to determine whether Ang-1 and Ang-2 may be considered ideal biomarkers for use in malaria detection. Regarding PCT, it was reported to be higher in severe malaria compared with uncomplicated infections. However, testing is limited by inconsistency in results, so it is necessary to carry out further research and development regarding the application of PCT as a biomarker in detecting malaria.

## Figures and Tables

**Table 1:** *Plasmodium* species detected by current malaria biomarkers and the types of samples that can be used.

Biomarker	Species of *Plasmodium*	Notes	Type of Samples That Can Be Used	Reference
HRP2	*P. falciparum*	Specific to *P. falciparum*	Serum, plasma, cerebrospinal fluid, and urine	[64]
pLDH	All *Plasmodium* species	-	Blood	[64]
Hemozoin	All *Plasmodium* species	-	Blood	[72]
Aldolase	*P. vivax*, *P. falciparum*		Blood	[75,76]
pGDH	*P. vivax*, *P. falciparum*	-	Blood	[78,93]
CRP	-	Not specific regarding which *Plasmodium*	Blood	[84]
Ang-1 and Ang-2	*P. falciparum*	-	Endothelium and red blood cells	[87]
PCT	*P. falciparum*	-	Serum	[89]

**Table 2:** The Current Application of Biomarkers and Malaria Detection Method.

Biomarker	Detection Method	Sensitivity	LOD	Specificity	Notes
HRP2	PfHRP2 rapid diagnostic test method	96% for *P. falciparum*	-	Specificity of 96.6% and a false positive rate of 9.3% for diagnosis of *P. falciparum* infection	Only for detecting *P. falciparum*
	Ultra-sensitive PfHRP2 quantification using the mPOC immunoassay	20-fold more sensitive than malaria RDTs	0.18 ng/mL	Specific for *P. falciparum*	Has not been detected in other *Plasmodium* species
PLDH	LFA with paper-based micellar ATPS	-	1 ng/μL	Specific to the primary anti-pLDH antibody	Not specific regarding which *Plasmodium* species
	A highly sensitive aptasensor toward *Plasmodium* lactate dehydrogenase	-	1 pM and pLDH protein detected at 1 parasite/μL	Specific for *Plasmodium falciparum* and *Plasmodium vivax*	Has not been detected in other *Plasmodium* species
HRP2-PLDH	Antibody-free Dual-biomarker Rapid Enrichment Workflow (AnDREW)	±9-fold improvement for PLDH and ±11-fold improvement for HRP2	3.80 parasite/μL	Specific for *Plasmodium falciparum* and *Plasmodium vivax*	Has not been detected in other *Plasmodium* species
HRP2-Aldolase	PfHRP2/pan aldolase combination rapid diagnostic test method	96% for *Plasmodium falciparum* and 62.5% for non–*P. falciparum* malaria species	-	Specificity of 87.3% and a false positive rate of 27.1% for diagnosis of *Plasmodium falciparum* infection	Has not been detected in other *Plasmodium* species
Hemozoin	Magnetic surface-enhanced Raman spectroscopy (M-SERS)	-	10^−11^ M (<10 parasites/µL)	Specific for the detection of hemozoin	Not specific regarding which *Plasmodium* species
Aldolase	Rapid diagnostic tests in *P. vivax*	97.4% sensitivity for *P. vivax*	-	Specific for *Plasmodium vivax*	Has not been detected in other *Plasmodium* species
PGDH	Protein-induced fluorescence enhancement	-	2.85 nM in dilute serum sample	Specific in detection of PfGDH for *Plasmodium falciparum*	Has not been detected in other *Plasmodium* species
CRP	High-sensitivity C-reactive protein	Low parasitemia with 96.4% sensitivity Moderate parasitemia with 80.9% sensitivity High parasitemia with 97.4% sensitivity	-	Low parasitemia with 79.3% specificity Moderate parasitemia with 91.9% specificity High parasitemia with 93,1% specificity	Not specific regarding which *Plasmodium* species

## Data Availability

Data sharing is not applicable.
